# Autistic traits and social anxiety in children and adolescents: the mediating role of theory of mind and social adaptive behavior

**DOI:** 10.1038/s41390-026-04791-1

**Published:** 2026-01-28

**Authors:** Ingrid Zugey Galán Vera, Rachele Lievore, Irene C. Mammarella

**Affiliations:** Department of Developmental and Social Psychology, University of Padova, Padova, Italy

## Abstract

**Background:**

Autistic traits refer to cognitive and behavioral characteristics seen in the general population that resemble those of autism but are less severe. Higher levels of autistic traits may be related to higher levels of social anxiety, and some variables may influence the relationship between autistic traits and social anxiety. In the present study we aimed to investigate the association between autistic traits and social anxiety, considering the possible influence of theory of mind and social adaptive behavior.

**Methods:**

The study involved 300 Italian parents of children and adolescents between 6 and 18 years old without any diagnosed medical or neurodevelopmental condition. Parents were asked to answer an online survey composed by different questionnaires on their children’s traits and abilities.

**Results:**

Our results suggest that autistic traits have a positive correlation with social anxiety and that theory of mind may act as a mediator in that relationship.

**Conclusion:**

We discussed as clinical and educational practice should prioritize training in perspective-taking skills to help preventing negative outcomes in children and adolescents.

**Impact:**

- Autistic traits and social anxiety seem to be associated in non-clinical population.
- Theory of mind may mediate the association between autistic traits and social anxiety.
- Educational interventions should focus on enhancing skills such as theory of mind.

## Introduction

Autism spectrum disorder (ASD) is characterized by challenges in social interaction and communication, as well as repetitive, limited and/or stereotyped patterns of behavior, activities and interests.^1^ However, ASD falls at the higher end of a range of difficulties in social and communication behaviors, which are likely spread continuously across a spectrum and vary from moderately to strongly inherited.^2,3^ Autistic traits are patterns of thinking and behavior seen in the general population that are similar to those in autism but are not severe enough to fulfill the criteria for a clinical diagnosis.^4^ Multiple studies have shown that elevated autistic traits are linked to lower academic performance, behavioral issues, challenges in adapting to school, and problematic relationships with peers.^5–7^ Additional research has found that elevated autistic traits are associated with symptoms of depression and anxiety, as well as with non-suicidal self-injury, social-emotional challenges, hypomania and psychotic symptoms,^6,8–12^ as well as less adaptation, well-being and self-efficacy.^13,14^

Recent research has reported that social anxiety is among the most frequent negative emotional outcomes experienced by autistic individuals.^15–17^ The same pattern appears to hold true for individuals who exhibit high levels of autistic traits.^18^ Social anxiety is characterized by an excessive fear of one or multiple social situations where the individual might be subject of possible judgement or scrutiny by others, for example, when having a conversation, or giving a talk.^1^ Previously it was considered that it manifested exclusively in adults; however, current evidence indicates that it can be present from the early stages of life,^19^ with an estimation of 1–2% in children and 2–6% in adolescents.^20,21^ Certain studies indicate that social anxiety is related to a higher risk of developing other anxiety disorders^22^ and may heighten the risk of beginning substance use,^23,24^ besides other negative outcomes such as body dysmorphic disorder, depression and eating disorders.^25,26^

The connection between autistic traits and social anxiety has been explored in several different contexts and populations, showing a positive correlation between them.^27–29^ In a research with adults diagnosed with social anxiety disorder, 70% of them reported a clinically significant level of autistic traits, which in turn had a major impact on their social skills.^30^ However, due to the similarity in some symptoms of autism and social anxiety, these conditions often overlap, making it challenging to distinguish between them.^31,32^ For example, domains of nonverbal communication, empathy, restricted interests and rumination could act as strong predictors of social anxiety, and it has also been observed that many of the symptoms are similar to those of autism.^18,33^ Moreover, a study involving both autistic and non-autistic adolescents with similarly high levels of social anxiety found that factors such as intolerance to uncertainty, alexithymia, and sensory hypersensitivity played a mediating role in the link between autistic traits and anxiety symptoms across the entire sample.^34^ Until now, there is limited evidence regarding the connection between autistic traits and social anxiety in children and adolescents with neurotypical development.

It is worth noting that certain variables influence the association between autistic traits and social anxiety. In this regard, theory of mind has been defined as the ability to understand that other people have their own thoughts, beliefs, desires, intentions, and emotions that may differ from one’s own,^35,36^ and has been shown to help prevent the development of negative emotional outcomes, such as anxiety and distress. For example, in adults’ samples, it has been observed that social anxiety and social cognition could be linked, as social anxiety appears to be negatively correlated with the ability to recognize others’ emotions and theory of mind and empathic accuracy.^37,38^ In a longitudinal study, children with better theory of mind skills experienced greater acceptance by their peers in subsequent years, showing levels of anxiety decreasing over time.^39^ Another study discovered a significant positive correlation between social anxiety and autistic traits in university students, with this relationship being mediated by their ability to resolve social problems.^29^ Importantly, this association has been documented exclusively in adults so far, with no prior evidence available for children and adolescents.

Regarding the link between theory of mind and autistic traits, most of the studies was conducted with a sample diagnosed with ASD. For example, it was found that the autistic participants tend to exhibit significantly lower theory of mind skills compared to the comparison group.^40–43^ It has also been observed that theory of mind contributed to language skills and vice versa, specifically discourse skills, assessing natural language while interacting with a parent.^44^ In addition, Early theory of mind abilities, like joint attention and social referencing, appear to mediate the connection between social communication difficulties and the severity of anxiety symptoms in autistic children.^45^ Instead, high levels of social anxiety tend to lead to more intense and meaningful attributions to the mental states of others.^38^ It has also been observed that autistic individuals, despite having impaired theory of mind, may experience more or less social anxiety depending on how well they are able to compensate in social situations due to higher cognitive abilities.^46^ Up to now, there is limited understanding of the relationship between theory of mind and autistic traits in populations of non-autistic children and adolescents.

In addition to theory of mind, social adaptive behavior might contribute to understanding the link between autistic traits and social anxiety. Research in the general population has shown that children and adolescents with higher levels of social anxiety tend to be less popular and more often rejected by their peers.^47^ Increased social worries appear to be strongly associated with lower academic performance and more problematic coping behaviors, such as self-injury,^48^ suggesting poorer ability to adapt to situational demands. As regards autistic people, some evidence suggest that they may be more at risk of having problems in social adaptation.^49^

However, to the best of our knowledge, no prior research has explored the link between autistic traits and social anxiety while considering the potential role of theory of mind and social adaptive behavior in a sample of non-autistic children and adolescents.

## The present study

The aim of this study was to explore the link between autistic traits and social anxiety, considering the potential influence of theory of mind and social adaptive behavior in a population of children and adolescents aged 6–18 years. The specific objectives were: (a) to examine the relationship between autistic traits and social anxiety, while considering the effects of sex and age to identify potential changes in the relationship based on these variables; (b) to understand how theory of mind and social adaptive behavior are linked to autistic traits and social anxiety; and (c) to determine if they could mediate the relationship between autistic traits and social anxiety.

Considering previous studies,^29,30,33^ we expect a positive relationship between autistic traits and social anxiety, although these studies included autistic participants and/or clinically diagnosed with social anxiety disorder. Regarding the role of sex, we anticipate no differences in the relationship between autistic traits and social anxiety between boys and girls, as has been observed in previous studies.^27,50^ Furthermore, we hypothesized that there would be no significant differences in the relationship between autistic traits and social anxiety based on participants’ age, as previous studies have found no age-related effects.^17,34,51^

In addition to the above and considering the theoretical background, we expect a negative relationship between autistic traits with theory of mind and social adaptive behavior, with higher autistic traits being related to lower theory of mind and more difficulties in social adaptive behavior.^45,46^ We also expect to find a negative relationship between social anxiety with theory of mind,^37^ and social adaptive behavior.^48,52^

Finally, given the scarcity of research on the mediators between autistic traits and social anxiety, a more exploratory approach has been taken to form hypotheses based on the third objective (i.e., the mediating role of theory of mind and social adaptive behaviors). It is reasonable to assume that theory of mind could mediate the relationship between autistic traits and social anxiety, with lower levels of theory of mind being linked to the development of social anxiety in participants with higher autistic traits.^37,39,45,46^ Similarly, poor social adaptive behavior may serve as a risk factor for the development of social anxiety in participants with higher levels of autistic traits.^52–54^ Thus, we could expect a mediating role of both theory of mind and social adaptive behavior.

## Method

### Participants

The sample included 300 Italian parents of children and adolescents between 6 and 18 years old (M_age_ = 10.69, SD = 3.25, 50% females) without any diagnosed medical or neurodevelopmental condition. Parents were asked to answer an online survey composed by different questionnaires on their children’s traits and abilities. 193 (around 64%) children and adolescents had older or younger siblings, and 231 (around 77%) lived in an urban area. 215 mothers and 85 fathers filled in the survey. Mothers of children and adolescents had a mean age range of 42.66 years old (SD = 6.47, min = 21, max = 66), and fathers 45.94 (SD = 9.67, min = 22, max = 71). 17 (5.6%) mothers had 8 years of formal education, 97 (32.3%) had 13 years of formal education, 59 (19.6%) had 16 years and 127 (42.3%) mothers at least 18 years; 29 (9.6%) fathers reported 8 years of formal education, 125 (41.6%) had 13 years, 40 (13.3%) had 16 years and 106 (35.3%) fathers 18 years or more years of formal education.

### Materials

The online survey involved a socio-demographic questionnaire in the initial part of the survey (12 items), including the following information related to the child: sex (male, female), date of birth, any medical conditions and neurodevelopmental disorders, age of first diagnosis if any, any medication. Moreover, in this initial part of the survey, parents were also asked who was answering the survey (mother, father), date of birth of both parents, education degree of both parents, whether they have other children, and whether they live in a rural or urban area. The entire survey included 207 items and was composed by several parent-reports which assessed the dimensions described in the following sections.

#### Autistic traits

The Social Responsiveness Scale (SRS;^55^) is a 65-item questionnaire, which can be completed by parents or teachers, and measures social communication and repetitive and restrictive behaviors that are related to autistic traits in both diagnosed children and the general population. For the purpose of this study, the parents’ version was used. It includes 5 subscales: social communication, social cognition, social motivation, social awareness, and autistic mannerisms. Parents were asked to choose the response that best described their child’s behavior over the past six months, each of the items is evaluated on a Likert scale ranging from 0 (“not true”) to 3 (“almost always true”). Some examples of the items are: “Avoids eye contact or has unusual eye contact”, “Shows unusual sensory interests (such as putting objects in the mouth or spinning them) or strange ways of using toys”. This test can be scored up to 195, with higher scores indicating more autistic traits. Raw scores were converted in standardized scores (T scores) distinguished by sex, and in each of the subscale, as well as an overall score. T-scores above 75 indicate a severe, clinically significant level of impairment in daily social interaction, strongly associated with a high autistic traits, while T-scores between 60 and 75 indicate a clinically significant reciprocal social behavior deficit that interferes with daily social interaction in a mild or moderate manner. On the other hand, scores equal to 59 or lower suggest the absence of autistic traits. The Italian adapted version,^56^ obtained a high internal consistency in a sample of the general population, also obtained a good test-retest reliability. In our sample, an acceptable omega value (Ω = 0.75, C.I. = 0.71–0.79) and a good Cronbach’s alpha (α = 0.81, C.I. = 0.78–0.84) was obtained.

#### Social anxiety

The Social Phobia and Anxiety Inventory for Children (SPAI-C-P;^57^) is a 26-item questionnaire that evaluates social anxiety levels in youth. Children are require to indicate how often they experience anxiety in specific social situations that could potentially trigger anxiety (e.g., reading aloud in class, performing in a play, eating in the school cafeteria), and assesses physical and cognitive characteristics of social phobia as well as avoidance behaviors. The parents’ version is identical to the children, except the stem of each item starts with e.g. “My child is afraid of being the center of attention” rather than “I’m afraid of being the center of attention”. Each of the 26 items is rated on a 3-point scale (1 = “never or hardly ever”, 2 = “sometimes”, 3 = “most of the time or always”) as was previously done in other studies.^58,59^ The maximum score is 78, higher scores indicate a higher level of social anxiety and worries. The SPAI-C has a good internal consistency and a significant correlation with the self-report version for children (Cronbach’s α = 0.94,^60^; Cronbach’s α = 0.90,^61^). In our sample, an excellent omega value (Ω = 0.91, C.I. = 0.9–0.93) as well as Cronbach’s alpha (α = 0.91, C.I. = 0.9–0.92) has been obtained.

#### Theory of mind

The Theory of Mind Inventory (ToMI;^62^) is a 48-item questionnaire in which parents answer in a form of report on their children, which aims to measure how capable their children are to infer the mental states of others. For the purpose of this study 29 items were considered, those related to the first order of beliefs, which is an ability that appears around the age of 4, referring to first-level recursive thinking (i.e., “I think that you think”). The items are Likert-type, ranging from 1 (“definitely not”) to 5 (“definitely”) points, in which parents must choose the number that most closely resembles their child’s behavior, some examples of the items are: “My child understands that when people get what they want they are happy”, “My child understands that it is important to hide well when playing hide and seek”. Considering the items of the first order, the maximum score is 145, and higher scores are an indicator of a greater ability to infer the mental states of others. The results obtained in the creation of this test showed good internal consistency and reliability. In our sample, we obtained an excellent omega value (Ω = 0.93, C.I. = 0.91–0.95) as well as Cronbach’s alpha (α = 0.93, C.I. = 0.92–0.94).

#### Social adaptive behavior

The Adaptive Behavior Assessment System – second edition (ABAS-2;^63^) is a 231-item rating system for evaluating adaptive behavior, ranging from 0 (“not able”) to 3 (“always/almost always”), which covers from birth to 89 years of age, with the purpose to assess the skills of a person in different areas of daily life to determine if he/she is able to function in different contexts without assistance. It includes three different domains: conceptual (communication, academic skills and self-direction), social (leisure time and socialization) and practical (use of community resources, home life, health and safety and self-care). In this study, only the social domain, which includes the subscales of socialization (number of items = 23) and leisure time (number of items = 22), was considered. This domain is composed of 45 items, examples of the items are: “Plans leisure activities in advance on free days or evenings”, “Has a stable group of friends”. The Italian version was adapted and standardized by Ferri et al.^64^, and reliability was α = 0.93. In our sample, we obtained an excellent omega value (Ω = 0.92, C.I. = 0.90–0.93) as well as Cronbach’s alpha (α = 0.91, C.I. = 0.90–0.93).

#### Procedure

This study was approved by the Ethics Committee of the University of Padova and took place between December 2022 and July 2023 and was carried out following the principles of the Declaration of Helsinki. The survey was shared through social networks and was conducted through the Qualtrics platform which parents could access via a link or QR code. At the beginning of the survey, informed consent was given by parents to participate in the project. Participants responded without obtaining any financial or other type of retribution. The average time to respond to the questionnaire was between 20 and 25 min, with no time limit for completion, having the possibility to complete the survey in different moments. No personal data were asked, and if parents wished, they had the possibility to write an email to receive a descriptive report of the raw results upon completion of the study.

#### Statistical approach

After checking the reliability of each measure in our sample, Pearson’s correlations were run as preliminary analyses to assess the relationships between autistic traits, social anxiety, theory of mind and social adaptive behavior. In addition, a regression model was performed to evaluate the moderating effect of age and sex with autistic traits in predicting social anxiety, to be able to detect any changes in the association between autistic traits and social anxiety depending on age and sex.

Then, the possible role of theory of mind and social adaptive behavior in the association between autistic traits and social anxiety was examined. For this purpose, a multiple mediation analysis was performed considering autistic traits as predictor, social anxiety as outcome, theory of mind and social adaptive behavior as mediators. We employed Preacher & Hayes’^65^ bootstrapping technique to compute both direct and indirect effects, utilizing 5000 bootstrap resamples. The significance of the indirect effect was determined by obtaining 95% confidence intervals (CIs). If these intervals do not encompass zero, the indirect effect is deemed statistically significant at the *p* < .05 threshold. Goodness of fit indexes^66^ were considered to assess the adequacy of the proposed mediation model: Comparative Fit Index (CFI; values closer to 1 indicate better fit), Tucker-Lewis Index (TLI; values closer to 1 indicate better fit), Root Mean Square Error of Approximation (RMSEA; values less than 0.06 typically indicate a good fit), and Standardized Root Mean Square Residual (SRMR; values less than 0.08 typically indicate a good fit). The same mediation analysis was then performed including the age as a covariate, and the best model was chosen based on fit indexes (see Supplementary Results, Supplementary Fig. S1).

Data were analyzed using R version 4.3.2.^67^ The following R packages were used: “stats”^67^ to perform the correlations and linear regression model, and “lavaan”^68^ for the mediation analysis.

## Results

### Preliminary analysis

Autistic traits show a significant positive correlation with social anxiety, *r* = 0.56, *p* < 0.001, and a significant negative correlation with social adaptive behavior, *r* = −0.49, *p* < 0.001 and theory of mind, *r* = −0.55, *p* < 0.001. Social anxiety is negatively correlated with social adaptive behavior, *r* = −0.35, *p* < 0.001, and theory of mind, *r* = −0.22, *p* < 0.001, while social adaptive behavior and theory of mind are positively correlated *r* = 0.32, *p* < 0.001. Table 1 displays the Pearson’s correlations between all variables, as well as the mean and standard deviation of each measure for this sample.

**Table 1:** Descriptive statistics and Pearson’s correlations between the variables of interest.

Variables	1	2	3	4
1. Autistic traits				
2. Social anxiety	0.56			
3. Theory of mind	−0.55	−0.22		
4. Social adaptive behavior	−0.49	−0.35	0.32	
*M (SD)*	54.34 (11.08)	38.28 (8.24)	130.93 (12.00)	88.00 (14.35)

All correlations are statistically significant with *p* < 0.001.

*M* mean, *SD* standard deviation.

A linear regression model with a three-way interaction was performed to evaluate any changes in the association between autistic traits and social anxiety depending on age and sex. The main effect of autistic traits was statistically significant in predicting social anxiety, *F* = 143.19, *p* < 0.001. However, no other statistically significant effects emerged for each factor (age: *F* = 1.85, *p* = 0.17; sex: *F* = 3.50, *p* = 0.07) and their interaction with autistic traits (autistic traits*age: *F* = 2.95, *p* = 0.09; autistic traits*sex: *F* = 2.09, *p* = 0.15; age*sex: *F* = 3.72, *p* = 0.06; autistic traits*age*sex: *F* = 1.61, *p* = 0.21) in predicting social anxiety.

### Multiple mediation analysis

Multiple mediation analysis included social anxiety as a dependent variable, autistic traits as a predictor, and theory of mind and social adaptive behavior as mediators. The direct effects of autistic traits on social anxiety (path c’), *b* = 0.44 [*bootstrap CI* = 0.33, 0.55], *SE* = 0.05, *z* = 9.48, *p* < 0.001, theory of mind (path a), *b* = −0.59 [*bootstrap CI* = −0.72, −0.46], *SE* = 0.05, *z* = −11.30, *p* < 0.001, and social adaptive behavior (path d), *b* = −0.63 [*bootstrap CI* = −0.75, −0.51], *SE* = 0.05, *z* = −9.67, *p* < 0.001, were statistically significant. Also, the direct effect of theory of mind on social anxiety (path b) was significant, *b* = 0.09 [*bootstrap CI* = 0.01, 0.19], *SE* = 0.04, *z* = 2.44, *p* = 0.01, whereas the link between social adaptive behavior and social anxiety (path e) was not significant, *b* = −0.06 [*bootstrap CI* = 0.01, 0.19], *SE* = 0.03, *z* = −1.94, *p* = 0.05. The indirect effect of theory of mind on the association between autistic traits and social anxiety was statistically significant, a*b: *b* = −0.06 [*bootstrap CI* = −0.11, −0.01], *SE* = 0.02, *z* = −2.38, *p* = 0.02. On the other hand, the indirect effect of social adaptive behavior on the link between autistic traits and social anxiety was not statistically significant, d*e: *b* = 0.04 [*bootstrap CI* = −0.01, 0.08], *SE* = 0.02, *z* = 1.90, *p* = 0.06. The total indirect effect obtained by summing the two indirect paths, was also not significant, ab+de: *b* = −0.02 [*bootstrap CI* = −0.09, 0.06], *SE* = 0.03, *z* = −0.57, *p* = 0.56. However, when considering the whole model, the total effect obtained by adding the direct effect of autistic traits on social anxiety with the indirect effects, was statistically significant, c’+abde: *b* = 0.41 [*bootstrap CI* = 0.34, 0.49], *SE* = 0.04, *z* = 11.80, *p* < 0.001. The fit indexes of the total model were excellent (CFI = 0.99, TLI = 0.99, RMSEA = 0.04, SRMR = 0.02). When age was added as a covariate in the model, the fit indexes were not acceptable (see Supplementary Results, Supplementary Fig. S1), so we decided to keep the model without the age as the best-fitting model.

**Fig. 1:**
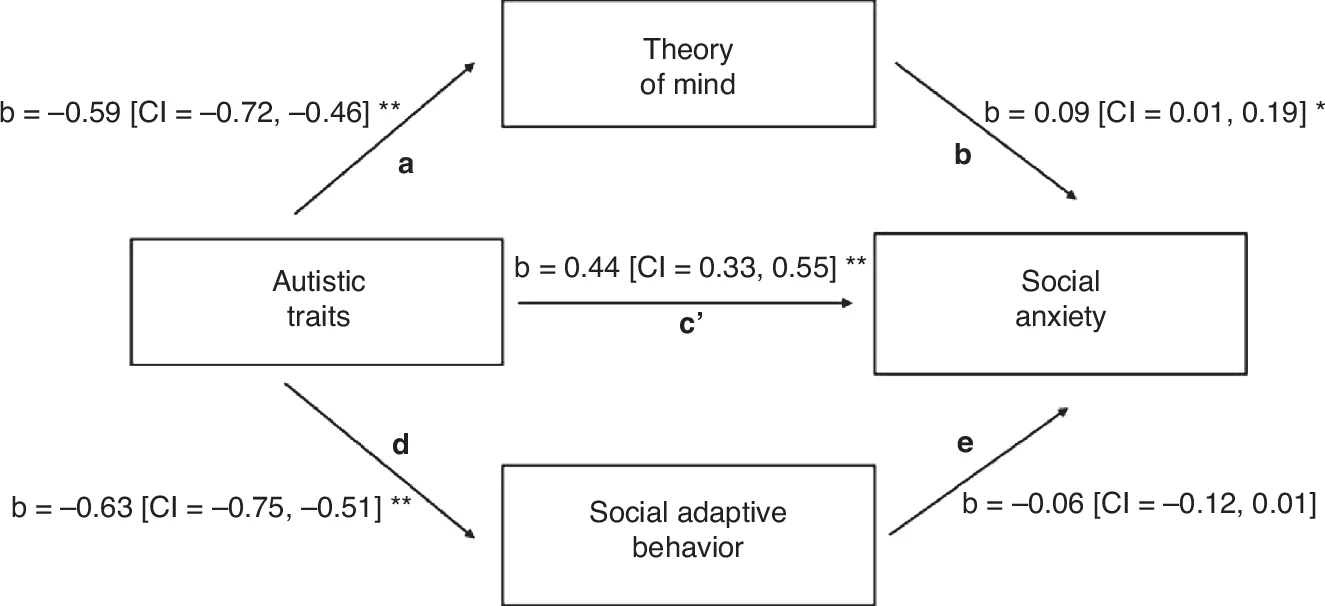
Multiple mediation model with social anxiety as the dependent variable, autistic traits as the predictor, and theory of mind and social adaptive behavior as mediators. Regression coefficients (b) and 95% bootstrap confidence intervals are shown for each path. Significant paths are indicated with asterisks (*p* < .05, *p* < 0.01).

Figure 1 shows the paths and the coefficients obtained from the multiple mediation model with social anxiety as the dependent variable, autistic traits as predictor, theory of mind and social adaptive behavior as mediators.

## Discussion

The primary objective of this study was to examine the relationship between autistic traits and social anxiety in children and adolescents aged 6–18, taking into account the potential mediating effects of theory of mind and social adaptive behavior.

In relation to our first objective concerning the connection between autistic traits and social anxiety, we identified a significant positive correlation between these variables, indicating that higher levels of autistic traits are associated with increased social anxiety. These findings align with those reported in earlier studies.^27–29^ Besides the well-known relationship between autism and social anxiety,^32,54^ these results consistently demonstrate a robust correlation between autistic traits and social anxiety symptoms across non-clinical populations, in which symptoms such as restricted interests, rumination, and social difficulties could also be observed.^29,69^ Additionally, when considering clinical populations, sex and age differences have been found, being more common to find higher levels of social anxiety in girls^70^ and older children,^71^ as well as a higher prevalence of autistic symptoms in boys.^72,73^ However, when considering non-clinical populations, consistent with previous studies, our findings showed no significant interaction effects of sex^27,50^ nor age^34,51^ with autistic traits in determining social anxiety. Nevertheless, previous research has noted that autistic traits may decrease with age, although their impact on social anxiety can remain stable.^74,75^ This underscores the importance of studying subclinical autistic traits and support the relevance of intervention research to better understand its relationship with social anxiety.

With respect to our second aim, which explored the relationship between theory of mind, adaptive social behavior, autistic traits, and social anxiety, significant correlations were identified in our sample. Specifically, for theory of mind, our findings demonstrated a strong negative correlation with autistic traits, indicating that higher autistic traits in neurotypically developing children are linked to lower theory of mind abilities. This result is consistent with previous findings observed in autistic individuals.^40,41,43^ Additionally, a significant negative correlation was observed between theory of mind and social anxiety. These findings align with earlier studies that also reported a negative association between theory of mind and social anxiety, likely because elevated social anxiety may cause individuals to misinterpret others’ thoughts and behaviors.^37–39^

Concerning social adaptive behavior, our findings revealed a significant negative correlation with autistic traits, which means that higher levels of autistic traits may be linked to difficulties in social adaptive behavior in the general population, as previously documented in autistic populations, in which the presence of difficulties in interacting and relating to others has been recurrently observed.^49^ Moreover, social adaptive behavior also negatively correlated with social anxiety, as found in previous studies in which children with higher levels of social anxiety were more rejected by their peers and were less popular,^47^ in addition to showing a lower academic performance and more problematic coping behaviors,^48^ which may hinder their social adaptation.

As regards our third aim on the possible mediating model of theory of mind and social adaptive behaviors in the association between autistic traits and social anxiety, some interesting insights emerged from the analyses. A significant indirect negative effect of theory of mind on the relationship between autistic traits and social anxiety was observed, suggesting that theory of mind may act as a mediator between these two variables. This result implies that participants with varying levels of autistic traits may also report differences in their social anxiety. Specifically, those with more autistic traits may have a lower theory of mind (*path a* is negative), which in turn leads to reduced social anxiety (*path b* is positive). Children and adolescents with more autistic traits may find difficulties in understanding other people’s beliefs and intentions.^40,41,43^ As autistic traits increase, theory of mind decreases, which could be associated with lower levels of social anxiety because individuals may be less aware of or concerned with social judgments.^76^ Instead, participants with lower autistic traits may experience higher levels social anxiety due to a better theory of mind, because they are more aware of social cues and potential judgments, which can heighten anxiety. In fact, stronger abilities to interpret the mental and emotional states of others may be associated with increased awareness of social dynamics, which could, in turn, lead to heightened levels of social anxiety.^38^

Furthermore, the indirect influence of social adaptive behavior on the association between autistic traits and social anxiety was found to be non-significant, suggesting that, social adaptive behavior may not act as a substantial mediator in the relationship between these variables. Indeed, cognitive abilities, particularly theory of mind, might have a greater influence on the emotional outcomes of children and adolescents than behavioral factors, such as maladaptive social behaviors.^77,78^ Nevertheless, dysfunctional social behaviors may become more important in determining negative emotional reactions and the development of social anxiety later in life.^79^ When taking into account the combined indirect effects of theory of mind and social adaptive behavior, the model reached significance, indicating that these two factors may jointly influence the relationship between autistic traits and social anxiety. Additionally, a direct positive association was found between autistic traits and social anxiety, implying that, regardless of theory of mind, individuals with higher autistic traits are more likely to experience elevated levels of social anxiety. In fact, those participants with higher autistic traits may also have difficulties within social contexts, which is a barrier to effective social interaction,^49^ and this in turn may cause emotional discomfort leading to higher worries in social contexts.^28,52,80^ In summary, although autistic traits are directly associated with higher social anxiety, this relationship is partially mitigated by a reduction in theory of mind. This suggests that the overall effect on social anxiety is a balance between the direct increase due to autistic traits and the indirect decrease resulting from lower theory of mind.

Although our findings are significant, certain limitations of the present study should be acknowledged. Firstly, since this study relies on a survey in which parents report on their children’s skills and behaviors, there is a potential for parents to overestimate, underestimate, or misinterpret these behaviors. This may be influenced for example by the level of attention they pay to their children, their personal parenting style, or the sociocultural context in which they are situated. Consequently, the survey results are based on parental perceptions, which inherently include various biases. Another limitation is that being an online survey, there is no guarantee that parents responded with accuracy or adequate attention, that they fully understood the instructions, or that they carefully read each item. Nevertheless, the data obtained appeared sufficiently consistent with previous findings and the measured showed high reliability.

Potential implications of the present research should target clinical and educational practise. The findings suggest that children and adolescents who exhibit autistic traits but do not meet the full diagnostic criteria for ASD,^4^ may also experience concerns and anxiety in social situations. This implies that they could present difficulties in interacting with others and establishing positive and healthy relationships, which in turn can influence social adaptation and generate mental health problems. Furthermore, the results underscore the potential benefits of interventions focused on enhancing skills such as theory of mind and social adaptive behavior,^81^ as these skills may be mediators of social challenges in children and adolescents with neurotypical development. Because until now there is no awareness of the need for children and adolescents with autistic traits to improve these skills, they are not provided with this type of attention.

In conclusion, autistic traits and social anxiety appear to be positively and significantly related even within non-clinical populations, with higher autistic traits linked to increased social anxiety levels. Furthermore, our findings indicate that theory of mind may serve as a mediator in the relationship between autistic traits and social anxiety among non-autistic children and adolescents, offering new avenues for exploring these variables in the general population.

## Supplementary information

Supplementary materials

## Data Availability

The datasets generated during and/or analyzed during the current study are available from the corresponding author on reasonable request.
