# Supplementary material for: Autistic traits and social anxiety in children and adolescents: the mediating role of theory of mind and social adaptive behavior
Source: Pediatr Res. 2026 Jan 28;100(3):1473–9. doi: 10.1038/s41390-026-04791-1 (PMC13672070; doi:10.1038/s41390-026-04791-1)

The association between autistic traits and social anxiety in children and adolescents: The mediating role of theory of mind and social adaptive behavior

1. Supplementary results

Multiple mediation analysis with age as a covariate

Multiple mediation analysis included social anxiety as a dependent variable, autistic traits as a predictor, theory of mind and social adaptive behavior as mediators, and age as a covariate in paths a, c' and d (see Figure S1).

The direct effects of autistic traits on social anxiety (path c'), *b*=.44 [*bootstrap CI*=.33, .55], *SE*=.05, *z*=7.98, *p*<.001, theory of mind (path a), *b*=-.51 [*bootstrap CI*=-.61, -.41], *SE*=.05, *z*=-10.33, *p*<.001, and social adaptive behavior (path d), *b*=-.76 [*bootstrap CI*=-.86, -.65], *SE*=.05, *z*=-13.84, *p*<.001, were statistically significant. Whereas the direct effect of theory of mind on social anxiety (path b) was not significant, *b*=.09 [*bootstrap CI*=.01, .19], *SE*=.04, *z*=1.7, *p*=.08, neither the association between social adaptive behavior and social anxiety (path e) *b*=-.03 [*bootstrap CI*=-.10, .03], *SE*=.03, *z*=-1.07, *p*=.28. The indirect effect of theory of mind on the relationship between autistic traits and social anxiety was not statistically significant, a*b: *b*=-.04 [*bootstrap CI*=-.10, -.01], *SE*=.02, *z*=-1.71, *p*=.08. Also the indirect effect of social adaptive behavior on the relationship between autistic traits and social anxiety was not statistically significant, d*e: *b*=.02 [*bootstrap CI*=-.02, .07], *SE*=.02, *z*=1.06, *p*=.28. The total indirect effect obtained by summing the two indirect paths, was also not significant, ab+de: *b*=-.02 [*bootstrap CI*=-.09, .05], *SE*=.03, *z*=-.51, *p*=.6. When considering the whole model, the total effect obtained by adding the direct effect of autistic traits on social anxiety with the indirect effects, was statistically significant, c'+abde: *b*=.42 [*bootstrap CI*=.35, .49], *SE*=.03, *z*=11.63, *p*<.001. However, the fit indexes of the total model were not good enough (CFI=.98, TLI=.83, RMSEA=.16, SRMR=.02).

Figure S1. Multiple mediation analysis with age as a covariate.

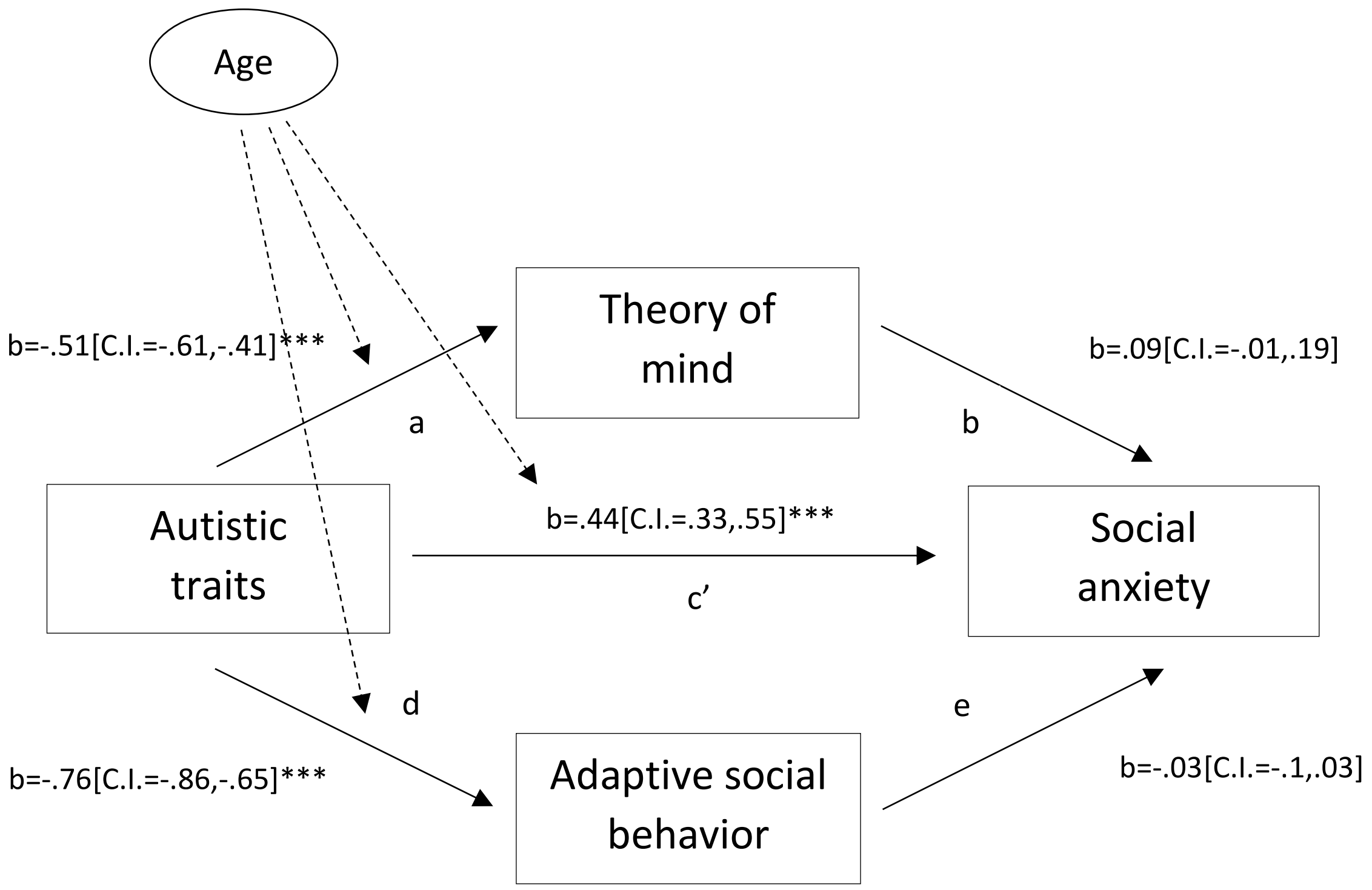

Supplement: Supplementary file 1 [file 41390_2026_4791_MOESM1_ESM.pdf]
